# Predisposing Factors Associated with Third-Generation Cephalosporin-Resistant *Escherichia coli* in a Rural Community Hospital in Thailand

**DOI:** 10.3390/antibiotics14080790

**Published:** 2025-08-04

**Authors:** Ratchadaporn Ungcharoen, Jindanoot Ponyon, Rapeepan Yongyod, Anusak Kerdsin

**Affiliations:** 1 Faculty of Public Health, Kasetsart University Chalermphrakieat Sakon Nakhon Province Campus, Sakon Nakhon 47000, Thailand; rapeepan.y@ku.th (R.Y.); anusak.ke@ku.th (A.K.); 2 Thatphanom Crown Prince Hospital, Nakhon Phanom 48110, Thailand

**Keywords:** *Escherichia coli*, *Klebsiella pneumoniae*, multidrug resistance, MDR

## Abstract

Background: Various predisposing factors contribute to the emergence and dissemination of the multidrug-resistant (MDR) phenotype in *Escherichia coli* and *Klebsiella pneumoniae*. Understanding these factors is crucial for guiding appropriate antimicrobial therapy and infection control strategies. This study investigated the predisposing factors contributing to the MDR characteristics of *E. coli* and *K. pneumoniae* isolated in a community hospital in northeastern Thailand. Methods: This case–control study utilized retrospective data from bacterial culture, as well as demographic, clinical, and antibiotic susceptibility records collected during 5 years (January 2016–December 2020). *E. coli* and *K. pneumoniae* isolates were analyzed from various clinical samples, including blood, urine, pus, sputum, and other body fluids. Data were analyzed using descriptive statistics and univariate logistic regression. Results: In total, 660 clinical isolates were analyzed (421 *E. coli* and 239 *K. pneumoniae*). Blood was the most common source of the detection of *E. coli* (63.0%) and sputum was the most common source of *K. pneumoniae* (51.0%). The median ages of patients were 67 and 63 years for *E. coli* and *K. pneumoniae*, respectively. *E. coli* cases were significantly associated with prior antibiotic use (OR = 1.79, 95% CI: 1.17–2.74 *p* = 0.008). MDR was observed in 50.1% of *E. coli* and 29.7% of *K. pneumoniae* (*p* < 0.001). *E. coli* compared to *K. pneumoniae* had lower resistance to third-gen cephalosporins (64.9% versus 95.8%) and carbapenems (8.0% versus 6.9%). ICU admission was the only factor significantly associated with MDR *E. coli* (OR = 2.40, 95% CI: 1.11–5.20 *p* = 0.026). No significant differences were observed in gender, age, or comorbidities between MDR cases. Antibiotic usage patterns also differed, with *E. coli* more likely to receive third-gen cephalosporins compared to carbapenems (OR = 3.02, 95% CI:1.18–7.74 *p* = 0.021). Conclusions: The use of third-generation cephalosporin may drive MDR *E. coli* more than *K. pneumoniae*. Prior antibiotic exposure was linked to *E. coli* bloodstream infections, while MDR *E. coli* showed greater clinical severity. These findings highlighted the need for improved antibiotic stewardship in rural hospitals.

## 1. Introduction

Multidrug-resistant (MDR) microorganisms, such as bacteria and fungi, have the ability to resist multiple classes of antimicrobial agents. According to the World Health Organization (WHO), a pathogen is classified as MDR when it becomes resistant to at least one agent in three or more antimicrobial categories. Multidrug resistance poses a critical public health challenge, particularly with *Escherichia coli* (*E. coli*) and *Klebsiella pneumoniae* (*K. pneumoniae*), which increasingly exhibit resistance to multiple drug classes. Antimicrobial resistance (AMR) is one of the most critical global health challenges today. Recognized by the WHO as a top 10 global health threat, AMR contributes to increased morbidity, mortality, and healthcare costs worldwide. The escalating burden of AMR is driven by factors such as poor infection control measures, inappropriate antibiotic use, and limited availability of novel antimicrobial agents [1]. Antibiotic-resistant *E. coli* and *K. pneumoniae* represent some of the most critical drug-resistant bacterial threats. As members of the Enterobacteriaceae family, these pathogens are of particular clinical importance due to their ability to cause infections across all age groups [2].

*E. coli* and *K. pneumoniae* represent the main members of the Gram-negative bacteria that constitute part of the normal human gastrointestinal microbiota. These organisms are recognized as substantially human-based pathogens responsible for a broad spectrum of both hospital-acquired and community-acquired infections, including diarrhea, urinary tract infections (UTIs) [3], bloodstream infections, and various other clinical manifestations [4,5,6]. *K. pneumnoniae*, a predominantly hospital-acquired opportunistic pathogen, accounts for approximately 30% of all Gram-negative bacterial infections [7]. This organism is associated with diverse clinical presentations, including bloodstream infections, pneumonia, UTIs, meningitis, pyogenic liver abscesses, and wound infections [3,8,9].

The Ministry of Public Health in Thailand [10] reported there are more than 100,000 patients with antimicrobial-resistant infections annually, while more than 30,000 patients die from antimicrobial-resistant infections, and the associated economic loss exceeds THB 40 billion, or approximately 1% of the country’s Gross Domestic Product (GDP) [11]. Commonly, Thai people are able to access antibiotics without prescriptions, such as through buying drugs from private drugstores or pharmacies, including small grocery stores in the community [12]. Inappropriate antibiotic self-medication remains widespread in Thailand, with studies showing most residents use antibiotics without prior professional consultation. Primarily, this practice involves drugs purchased over the counter or reused from previous prescriptions, often for conditions that do not require antibiotics [5,12].

Epidemiological data assessing multidrug-resistant Enterobacteriaceae (MDRE), specifically *Escherichia coli* and *Klebsiella pneumoniae*, across various global regions have demonstrated a significant increase in prevalence, with notable geographical variation among countries [13]. The emergence and dissemination of multidrug-resistant *E. coli* are influenced by multiple interrelated factors. Prior antibiotic use (particularly inappropriate or excessive administration) creates selective pressure favoring resistant strains in both clinical and agricultural settings [14,15,16]. The extensive use of antibiotics in animal husbandry, especially as growth promoters, further exacerbates antimicrobial resistance by fostering resistant bacteria transmissible to humans [16,17,18]. Environmental contamination, including the presence of resistant *E. coli* in water sources, plays a critical role in spreading resistance [19,20,21]. In healthcare settings, prolonged hospitalization, intensive care unit (ICU) admissions, and invasive devices such as urinary catheters significantly increase the risk of MDR *E. coli* infections [22,23]. Patient-related factors, such as advanced age, female sex, and comorbidities (such as diabetes mellitus), also contribute to increased susceptibility [22,24]. Importantly, developing countries face a disproportionately high burden of MDR infections, due to limited healthcare infrastructure, inadequate infection control, poor sanitation, and restricted access to clean water [24,25]. Recent evidence has also drawn attention to coinfection-related factors as important contributors to the emergence and persistence of multidrug resistance. Sophonsri et al. [26] reported that coinfection with carbapenem-resistant *Pseudomonas aeruginosa* or *Acinetobacter baumannii* frequently occurred in patients infected with carbapenem-resistant *Klebsiella pneumoniae*, particularly among those with prior carbapenem exposure and a history of pneumonia. In a separate study, Lv et al. [27] identified fiberoptic bronchoscopy, repeated blood transfusion, and previous use of tigecycline as significant risk factors for coinfection involving carbapenem-resistant *Klebsiella pneumoniae* and *Acinetobacter baumannii*.

This study investigated the predisposing factors for antimicrobial resistance and the MDR characteristics of *E. coli* and *K. pneumoniae* in a community hospital serving a rural population in northeastern Thailand. While the epidemiology of MDR Enterobacteriaceae has been well-studied globally, most data come from urban or tertiary hospitals in high-income countries, which may not reflect rural, resource-limited settings. In rural Thailand, factors such as unregulated antibiotic use, limited diagnostics, inconsistent stewardship, and local health behavior may uniquely drive resistance. Often, community hospitals lack surveillance and infection control programs. Therefore, the current study should fill a crucial gap by identifying risk factors specific to this context, aiming to guide tailored interventions against antimicrobial resistance in rural Thai communities.

## 2. Results

### 2.1. E. coli and K. pneumoniae Isolates by Specimen Type

In total, 660 clinical specimens were analyzed, consisting of 421 *E. coli* isolates and 239 *K. pneumoniae* isolates. The distribution of these isolates by specimen type is presented in Table 1. Among the *E. coli* isolates, the highest proportion was in blood (63.0%), followed by urine (26.8%). Sputum represented 5.9% of *E. coli* isolates, while pus samples accounted for 3.3%. Fluid specimens contributed 1.0%. For *K. pneumoniae*, in blood, urine, sputum, pus, and fluid, the rates cultured were 32.2%, 10.5%, 51.0%, 3.8%, and 2.5%, respectively. The age range of patients was 2–96 years. The median age of cases for *E. coli* was 67 (IQR = 22.0) years, and for *K. pneumoniae*, it was 63 (IQR = 19.5) years.

Among the 421 patients with *E. coli* isolates, 156 (37.1%) had bloodstream infections. Comorbidities included diabetes mellitus (DM), hypertension (HT), chronic obstructive pulmonary disease (COPD), and chronic kidney disease (CKD). Prior antibiotic use was significantly associated with bloodstream infection (OR = 1.79, 95% CI: 1.17–2.74, *p* = 0.008). Other factors were not significantly associated with *E. coli* bloodstream infection: gender (OR = 1.18; 95% CI: 0.77–1.80), age ≥ 60 years (OR = 1.47; 95% CI: 0.96–2.22), diabetes mellitus (OR = 1.58; 95% CI: 0.90–2.77), hypertension (OR = 0.66; 95% CI: 0.39–1.16), COPD (OR = 0.47; 95% CI: 0.12–1.83), CKD (OR = 0.62; 95% CI: 0.23–1.67), and ICU admission (OR = 1.89; 95% CI: 0.83–4.31) (Table 2).

### 2.2. Multidrug Resistance Patterns of E. coli and K. pneumoniae

Multidrug resistance patterns were observed in 50.1% of *E. coli* (*n* = 221) and 29.7% of *K. pneumoniae* (*n* = 71) isolates, which are subsets of the total isolates (421 and 239, respectively) identified in this study. The results of this analysis, presented in Table 3, revealed striking differences in resistance profiles between the two pathogens. Multidrug resistance was observed in 211 (50.1%) *E. coli* and 71 (29.7%) *K. pneumoniae* isolates (*p*-value < 0.001, OR = 2.38, 95% CI: 1.70–3.33). There were significant differences in third-gen cephalosporin (*p*-value < 0.001) and carbapenem (*p*-value < 0.001) resistance between the two pathogens. The antibiotic susceptibility patterns among the MDR strains are shown in Table 3.

Table 4 presents the distribution of antibiotic resistance between *Escherichia coli* and *Klebsiella pneumoniae* and their associated odds ratios (ORs). Comparing the use of third-generation cephalosporins to aminoglycosides and quinolones, *E. coli* was more likely to be resistant to third-generation cephalosporins than *K. pneumoniae*, although there was no significant difference (OR = 0.69, 95% CI: 0.48–1.00, *p* = 0.052). Among the total of 577 cases, *E. coli* was resistant to third-generation cephalosporins in 137 out of 414 strains (33.1%), whereas *K. pneumoniae* was resistant in 68 out of 163 strains (41.7%).

In contrast, comparing the use of third-generation cephalosporins to carbapenems, *E. coli* had a significantly higher likelihood of being resistant to cephalosporins (OR = 3.02, 95% CI: 1.18–7.74, *p* = 0.021) compared to *K. pneumoniae*. In a subgroup of 225 cases, *E. coli* was resistant to third-generation cephalosporins in 137 out of 145 cases (94.5%), whereas *K. pneumoniae* was susceptible in 68 out of 80 cases (85.0%). These results demonstrate statistically significant differences in antibiotic resistance between *E. coli* and *K. pneumoniae*.

### 2.3. Analysis of Clinical Characteristics

Comparative analysis between MDR *E. coli* (*n* = 211) and *K. pneumoniae* (*n* = 71) isolates revealed several characteristics. Among MDR *E. coli* isolates, 112 (53.1%) were from female patients, while for MDR *K. pneumoniae*, 39 (54.9%) isolates were from male patients (OR = 1.38, 95% CI: 0.80–2.37, *p* = 0.24). Regarding age distribution, patients aged more than 60 years accounted for 57.8% (*n* = 122) of MDR *E. coli* cases and 52.1% of MDR *K. pneumoniae* cases (OR = 1.26, 95% CI: 0.73–2.16), *p* = 0.402), suggesting a higher prevalence of MDR distribution among elderly patients, although this difference was not statistically significant.

The comorbidities examined in this study, including diabetes mellitus (DM), hypertension (HT), chronic obstructive pulmonary disease (COPD), and chronic kidney disease (CKD), showed no statistically significant association with either type of MDR organism. Previous antibiotic received (Previous ATB) showed a trend toward association with MDR *E. coli* infection (OR = 1.790, 95% CI: 0.97–3.30), although this factor did not reach statistical significance (*p*-value = 0.063).

Importantly, ICU admission had a statistically significant association with MDR *E. coli* infection (OR = 2.40, 95% CI: 1.11–5.20, *p*-value = 0.026) compared to *K. pneumoniae*. This may suggest that MDR *E. coli* is associated with more severe causes (Table 5).

## 3. Discussion

MDR *E. coli* and *K. pneumoniae* pose a major threat in hospital settings, leading to increased morbidity, mortality, and healthcare costs. Understanding the predisposing factors associated with these infections is crucial for implementing effective prevention and control strategies. These factors range from patient demographics and underlying conditions to hospitalization-related exposures and the selective pressure of antibiotic use [28,29]. By identifying these risks, healthcare providers can target interventions to reduce the spread of MDR organisms and improve patient outcomes [30].

In the current study, prior antibiotic use was more strongly associated with MDR *Escherichia coli* than with MDR *Klebsiella pneumoniae*, which is consistent with another study that highlighted species-specific differences in resistance development and antibiotic selective pressures [10]. Typically, MDR *E. coli* demonstrates high resistance rates to commonly used antimicrobials, such as amoxicillin, cefuroxime, trimethoprim–sulfamethoxazole, tetracycline, and fluoroquinolones, with prior antibiotic exposure recognized as a significant risk factor for colonization or infection with resistant strains [31,32]. In particular, the prolonged or inappropriate use of fluoroquinolones has been linked to increased resistance in *E. coli* [31]. Mechanistically, prior antibiotic exposure has been shown to disrupt the intestinal microbiota, favoring the overgrowth and persistence of resistant *E. coli* strains, which may partly explain the stronger association observed in the current study [33].

In contrast, although it has been suggested that prior exposure to antibiotics, such as aminoglycosides, penicillins, colistin, or linezolid, may increase the risk of MDR *K. pneumoniae* infection, the overall relationship appears less consistent and is likely influenced by the combination and spectrum of the antibiotics administered [34]. Longitudinal comparative data support an overall rise in multidrug resistance among both *E. coli* and *K. pneumoniae* isolates, particularly from urinary tract infections; however, the strength and consistency of prior antibiotic use as a risk factor remains more prominent for *E. coli* [35,36].

Based on the results from the current study, admission to the ICU was significantly associated with *E. coli* infection compared to *K. pneumoniae*, with an OR of 2.403 (95% confidence interval (95% CI): 1.111–5.200), indicating that ICU patients were more than twice as likely to acquire *E. coli* infections than non-ICU patients. However, a study by Moini et al. [37] reported no statistically significant difference in clinical outcomes between drug-resistant and non-resistant *E. coli* infections. Furthermore, evidence from a study conducted in Thailand indicated that *E. coli* may have a higher infection rate in ICU settings [38]. In particular, the production of extended-spectrum beta-lactamases (ESBLs) and carbapenemases has been strongly associated with adverse clinical outcomes, including therapeutic failure and increased mortality rates [39]. The ICU environment itself is recognized as a high-risk setting for the emergence and transmission of multidrug resistance. Several contributing factors, such as invasive procedures, prolonged hospitalization, prior use of broad-spectrum antibiotics, and the critical condition of patients, create selective pressure that favors the survival of resistant strains (particularly *E. coli*), which remains a leading cause of urinary tract infections [40].

Determining whether MDR *E. coli* or MDR *K. pneumoniae* imposes a greater clinical burden in ICU patients remains inconclusive. The impact of each pathogen is influenced by a variety of complex factors, including specific resistance mechanisms, pathogen virulence, and individual patient characteristics. Furthermore, comparative studies between MDR *E. coli* and MDR *K. pneumoniae* are still relatively limited. Hospital size and setting also play important roles, as variations in clinical context and patient volume may affect infection patterns and outcomes. These considerations highlight the need for a comprehensive and systematic evaluation of existing evidence to accurately assess the relative clinical impact of each organism in terms of mortality, disease severity, and healthcare costs.

Several studies have supported the conclusion that resistance to third-generation cephalosporins differs significantly between *E. coli* and *K. pneumoniae*. The current study revealed that receiving cephalosporin tended to induce a greater level of MDR *E. coli* than of MDR *K. pneumoniae* compared to the use of carbapenem. Lee et al. [41] reported that the resistance rate to third-generation cephalosporins in *E. coli* was 22.8%, higher than the 19.6% observed in *K. pneumoniae*. In addition, the Australian Passive Antimicrobial Resistance Surveillance [42] noted increasing resistance trends in both bacteria, but with differing rates. Furthermore, Lee et al. [41] reported a significant association between antibiotic usage and resistance rates to third-generation cephalosporins in both *E. coli* and *K. pneumoniae*, with notable differences in resistance levels. Other studies have highlighted differing resistance mechanisms, such as the production of ESBLs and the dissemination of resistance genes in each species.

Conversely, one study reported no significant difference in third-generation cephalosporin resistance between *E. coli* and *Klebsiella* spp. For example, Manninen et al. [43] reported very low resistance rates to second- and third-generation cephalosporins in Finland, with no significant difference between *E. coli* and *Klebsiella* spp. Their study suggested that low antibiotic consumption and effective stewardship may have contributed to reduced resistance in both bacteria. Other studies have indicated that cephalosporin resistance may not always correlate directly with resistance to other antibiotic classes in certain clinical contexts.

In summary, resistance to third-generation cephalosporins can vary significantly between *E. coli* and *K. pneumoniae*, largely due to genetic mechanisms and the spread of resistance genes, such as ESBL production, which differ in type and frequency between the two species. Furthermore, differences in antibiotic usage patterns and stewardship across regions influence resistance rates. Understanding these differences is crucial for optimizing treatment strategies and implementing effective resistance control measures in both hospital and community settings [42,44,45].

This study has several limitations. First, the relatively small sample size may limit the generalizability of the findings. Second, as the data were collected from a single community hospital, the results may not be representative of the broader population, particularly in urban settings. Third, stratification of prior antibiotic use by specific antibiotic classes was not possible, as this information was not collected. This may have introduced bias, as different antibiotic classes vary in their potential to promote MDR colonization. Additionally, certain clinically relevant variables, such as corticosteroid use, length of hospital stay, and the presence of coinfection, were initially excluded from the analysis. Their omission may have confounded the association observed between prior antibiotic exposure and MDR *E. coli* infection. Nonetheless, this finding should be interpreted with caution. Future studies with a larger, multicenter design are recommended to improve external validity and better capture population diversity.

## 4. Materials and Methods

### 4.1. Study Design and Data Collection

This case–control study analyzed five-year retrospective data (1 January 2016–31 December 2020) of *E. coli* and *K. pneumoniae* isolates from clinical specimens at a 120-bed community hospital in northeastern Thailand. Only one isolate per case was enrolled. Statistical significance was determined using two-sided tests and 95% confidence intervals (95% CI).

The study compiled bacterial identification and antimicrobial susceptibility testing (AST) results from all patients admitted to a community hospital during the study period. Microbial laboratory analyses were performed at the provincial hospital with results stored in the MLAB database (Version 5.58.22.02.28). The same standardized protocols for culture, identification, and AST were consistently conducted, according to Laboratory Accreditation under The Association of Medical Technologist of Thailand. AST results were interpreted based on the Clinical and Laboratory Standards Institute (CLSI) guidelines and classified as susceptible, intermediate, or resistant [46]. The selection of diagnostic examinations, including blood, stool, or urine tests, was guided by the clinical presentation and the attending physician’s judgment for each patient. Diagnostic tests were ordered based on the suspected site of infection and relevant comorbidities. This approach ensured that diagnostic and susceptibility testing were appropriately tailored to the clinical context and individual patient conditions.

A cohort of designated patients was established, and relevant clinical factors were extracted from both physical medical records and electronic medical records maintained in the HOSxP system. Additionally, medical history during the study period—including prior antimicrobial exposure and comorbid conditions—was obtained from the electronic records. The hospital provides inpatient care across five divisions: pediatric ward, male ward, female ward, special ward, and intensive care unit (ICU). For analytical purposes, we categorized these as ICU and non-ICU settings, with the latter comprising the pediatric, male, female, and special wards. Since these wards were not organized according to physician specialties but rather for administrative purposes, antimicrobial resistance surveillance was necessary across all units due to the absence of specialized patient classification.

Cases were defined as patients with multidrug-resistant (MDR) *E. coli* infections, while controls were patients with MDR *K. pneumoniae* isolates demonstrating resistance to at least three antibiotic classes.

Patients were eligible for inclusion if they met the following criteria:

(1) Were admitted to Thatphanom Crown Prince Hospital for more than 24 h;
(2) Had positive microbial culture results for *Escherichia coli* or *Klebsiella pneumoniae*;
(3) Underwent antimicrobial susceptibility testing (AST).

Patients were excluded if they had

(1) Incomplete biological or geographic data;
(2) No recorded medical history at Thatphanom Crown Prince Hospital during the study period;
(3) A hospital stay of less than 24 h.

### 4.2. Statistical Analysis

Data were checked, cleaned, and double-entered into Microsoft Excel and then exported to Jamovi version 2.3.28. Descriptive statistics were used to characterize resistance patterns, with frequencies reported as percentages. Chi-square tests compared susceptibility rates between antibiotic groups in MDR isolates. Univariate logistic regression analyses examined clinical and demographic differences between *E. coli* and *K. pneumoniae* infections, with results expressed as ORs with 95% confidence intervals.

### 4.3. Ethical Approval

The study was approved by the Ethics Committee of Human Research Nakhon Phanom Provincial Health Office, Ministry of Public Health (Protocol Number REC 014/64).

## 5. Conclusions

This study suggests that the use of third-generation cephalosporins may be more strongly associated with the emergence of MDR *E. coli* than with *K. pneumoniae* in a community hospital setting, highlighting the possible influence of antimicrobial selection pressure in rural healthcare environments. Additionally, prior antibiotic use was linked to an increased risk of *E. coli* bloodstream infections. While MDR *E. coli* infections appeared to present with greater clinical severity—as reflected by higher ICU admission rates—these observations warrant further investigation in larger, multicenter studies. Overall, the findings support the need for context-specific antimicrobial stewardship and strengthened infection control strategies in community hospitals.

## Figures and Tables

**Table 1:** Descriptive summary of *E. coli* and *K. pneumoniae* isolates by specimen type.

Characteristic	*E. coli* Infection		Total	OR (95% CI)	*p*-Value
	Blood (*n* = 156)	Other (*n* = 265)			
Gender					0.569
Male	68 (43.6)	108 (40.8)	176	ref	
Female	88 (56.4)	157 (59.2)	245	1.18 (0.77–1.80)	
Age group					0.181
<60	71 (45.5)	103 (38.9)	174	ref	
≥60	85 (54.5)	162 (61.1)	247	1.47 (0.96–2.22)	
DM					0.353
No	125 (80.1)	202 (76.2)	327	ref	
Yes	31 (19.9)	63 (23.8)	94	1.58 (0.90–2.77)	
HT					0.42
No	119 (76.3)	211 (79.6)	330	ref	
Yes	37 (23.7)	54 (20.4)	91	0.66 (0.39–1.16)	
COPD					0.128
No	150 (96.2)	261 (98.5)	411	ref	
Yes	6 (3.8)	4 (1.5)	10	0.47 (0.12–1.83)	
CKD					0.341
No	147 (94.2)	255 (96.2)	402	ref	
Yes	9 (5.8)	10 (3.8)	19	0.62 (0.23–1.67)	
Previous ATB					0.008 *
Yes	65 (41.7)	77 (29.1)	142	1.79 (1.17–2.74)	
No	91 (58.3)	188 (70.9)	279	ref	
ICU					0.132
No	143 (91.7)	252 (95.1)	395	1.89 (0.83–4.31)	
Yes	13 (8.3)	13 (4.9)	26	ref	

*: *p*-value < 0.05, ref: reference; ATB: antibiotic; ICU: intensive care unit; DM: diabetes mellitus; HT: hypertension; CKD: chronic kidney disease; COPD: chronic obstructive pulmonary disease.

**Table 2:** Distribution of *E. coli* and *K. pneumoniae* by clinical specimens.

Specimen	*E. coli* (*n* = 421) *n* (%)	*K. pneumoniae* (*n* = 239) *n* (%)
Blood	265 (63.0)	77 (32.2)
Urine	113 (26.8)	25 (10.5)
Sputum	25 (5.9)	122 (51.0)
Pus	14 (3.3)	9 (3.8)
Fluid	4 (1.0)	6 (2.5)

**Table 3:** Antimicrobial susceptibility in clinical isolates of *E. coli* (*n* = 211) and *K. pneumoniae* (*n* = 71).

Antibiotic Type	*E. coli* (*n* = 211)		*K. pneumoniae* (*n* = 71)		*p*-Value
	R	S	R	S	
Aminoglycosides	117 (55.5)	94 (44.5)	36 (50.7)	35 (49.3)	0.487
Penicillin	210 (99.5)	1 (0.5)	71(100.0)	0 (0.0)	0.561
3rd-generation Cephalosporins	137 (64.9)	74 (35.1)	68 (95.8)	3 (4.2)	<0.001 **
Quinolones	160 (75.8)	51 (24.2)	59 (83.1)	12 (16.9)	0.203
Carbapenems	8 (3.8)	203 (96.2)	12 (6.9)	59 (83.1)	<0.001 **

**: *p*-value < 0.001, S: susceptible; R: resistant.

**Table 4:** Comparison of antimicrobial resistance between *Escherichia coli* and *Klebsiella pneumoniae*.

Antibiotic Type	*E. coli*	*K. pneumoniae*	Total	OR (95% CI)	*p*-Value
	R	R			
3rd generation Cephalosporins	137	68	205	0.69 (0.48–1.00)	0.052
Aminoglycosides and Quinolones	277	95	372	ref	
Total	414	163	577		
3rd generation Cephalosporins	137	68	205	3.02 (1.18–7.74)	0.021 *
Carbapenems	8	12	20	ref	
Total	145	80			

*: *p*-value < 0.05, R: resistant.

**Table 5:** Characteristics of MDR *E. coli* compared with MDR *K. pneumoniae* infections by univariate logistic regression analysis.

Characteristics	MDR		OR (95% CI)	*p*-Value
	*E. coli* (*n* = 211)	*K. pneumoniae* (*n* = 71)		
Gender				
Male	99 (46.9)	39 (54.9)	ref	0.244
Female	112 (53.1)	32 (45.1)	1.38 (0.80–2.37)	
Age group (years)				
<60	89 (42.2)	34 (47.9)	ref	
≥60	122 (57.8)	37 (52.1)	1.26 (0.73–2.16)	0.402
DM				
No	160 (75.8)	57 (80.3)	ref	
Yes	51 (24.2)	14 (19.7)	1.30 (0.67–2.52)	0.442
HT				
No	165 (78.2)	58 (81.7)	ref	
Yes	46 (21.8)	13 (18.3)	1.24 (0.62–2.47)	0.532
COPD				
No	206 (97.6)	68 (95.8)	ref	
Yes	5 (2.4)	3 (4.2)	0.55 (0.13–2.36)	0.422
CKD				
No	203 (96.2)	66 (93.0)	ref	
Yes	8 (3.8)	5 (7.0)	0.52 (0.16–1.65)	0.266
Previous ATB				
No	135 (64.0)	54 (76.1)	ref	
Yes	76 (36.0)	17 (23.9)	1.79 (0.97–3.30)	0.063
ICU				
No	193 (91.5)	58 (81.7)	ref	
Yes	18 (8.5)	13 (18.3)	2.40 (1.11–5.20)	0.026 *

*: *p*-value < 0.05; OR: odds ratio; 95% CI: 95% confidence interval; ref: reference. ATB: antibiotic used; ICU: intensive care unit; DM: diabetes mellitus; HT: hypertension; CKD: chronic kidney disease; COPD: chronic obstructive pulmonary disease.

## Data Availability

The datasets used and/or analyzed during the current study are available from the corresponding author upon reasonable request.

## Abbreviations

The following abbreviations are used in this manuscript:

MDR	Multidrug-resistant
MDRE	Multidrug-resistant Enterobacteriaceae
*E. coli*	*Escherichia coli*
*K. pneumoniae*	*Klebsiella pneumoniae*
OR	Crude odds ratio
ORadj	Adjusted odds ratio
95%CI	95% confidence interval
